# Scoring Enzootic Pneumonia-like Lesions in Slaughtered Pigs: Traditional vs. Artificial-Intelligence-Based Methods

**DOI:** 10.3390/pathogens12121460

**Published:** 2023-12-17

**Authors:** Jasmine Hattab, Angelo Porrello, Anastasia Romano, Alfonso Rosamilia, Sergio Ghidini, Nicola Bernabò, Andrea Capobianco Dondona, Attilio Corradi, Giuseppe Marruchella

**Affiliations:** 1 Department of Veterinary Medicine, University of Teramo, Loc. Piano d’Accio, 64100 Teramo, Italy; jhattab@unite.it; 2 AImageLab, University of Modena and Reggio Emilia, Via Vivarelli 10/1, 41125 Modena, Italy; 169084@studenti.unimore.it; 3 Associació Porcsa. GSP, Partida La Caparrella 97C, 25192 Lleida, Spain; anastasia.romano97@gmail.com; 4 Istituto Zooprofilattico Sperimentale della Lombardia e dell’Emilia-Romagna “Bruno Ubertini” (IZSLER), 25124 Brescia, Italy; alfonso.rosamilia@izsler.it; 5 Department of Food and Drug, University of Parma, Via del Taglio 10, 43126 Parma, Italy; sergio.ghidini@unipr.it; 6 Department of Bioscience and Technology for Food, Agriculture and Environment, University of Teramo, Via Renato Balzarini 1, 64100 Teramo, Italy; nbernabo@unite.it; 7 Farm4trade s.r.l., Via IV Novembre 33, 66041 Atessa, Italy; andrea@farm4trade.com; 8 Department of Veterinary Science, University of Parma, Via del Taglio 10, 43126 Parma, Italy; attilio.corradi@unipr.it

**Keywords:** slaughtered pigs, enzootic pneumonia, score, artificial intelligence

## Abstract

Artificial-intelligence-based methods are regularly used in the biomedical sciences, mainly in the field of diagnostic imaging. Recently, convolutional neural networks have been trained to score pleurisy and pneumonia in slaughtered pigs. The aim of this study is to further evaluate the performance of a convolutional neural network when compared with the gold standard (i.e., scores provided by a skilled operator along the slaughter chain through visual inspection and palpation). In total, 441 lungs (180 healthy and 261 diseased) are included in this study. Each lung was scored according to traditional methods, which represent the gold standard (Madec’s and Christensen’s grids). Moreover, the same lungs were photographed and thereafter scored by a trained convolutional neural network. Overall, the results reveal that the convolutional neural network is very specific (95.55%) and quite sensitive (85.05%), showing a rather high correlation when compared with the scores provided by a skilled veterinarian (Spearman’s coefficient = 0.831, *p* < 0.01). In summary, this study suggests that convolutional neural networks could be effectively used at slaughterhouses and stimulates further investigation in this field of research.

## 1. Introduction

Swine enzootic pneumonia (EP) is caused by *Mycoplasma hyopneumoniae* and represents a relevant component of the so-called porcine respiratory disease complex (PRDC). Except for a few European countries (e.g., Switzerland, Norway, and Finland), *M. hyopneumoniae* can still be considered a ubiquitous pathogen in pig farming worldwide. Ciliated cells, from trachea to bronchioles, are the main target of *M. hyopneumoniae* colonization, which causes cilia loss and epithelial cell disruption, thus predisposing to secondary viral and bacterial infections. The clinical onset and outcome of *M. hyopneumoniae* infection are usually insidious, chronic, slowly progressive, and strongly influenced by concurrent infections. Likewise, gross lesions remain visible for several months after the infection and appear as bilateral foci of bronchopneumonia affecting the cranio-ventral portion of lungs. Similar lesions can be caused by different pathogens (e.g., swine influenza virus) and are commonly defined as EP-like lesions [1,2,3].

There is no doubt that PRDC negatively impacts the profitability of pig farming. However, it is challenging to reliably estimate economic losses caused by EP at the farm level due to its main clinical features. In this respect, post mortem inspections at slaughter offer a unique opportunity to quantify EP effectively and efficiently in pig herds. In fact, EP-like lesions recover very slowly and are still present at slaughter, where they can be detected and scored [4,5,6].

**Figure 1:**
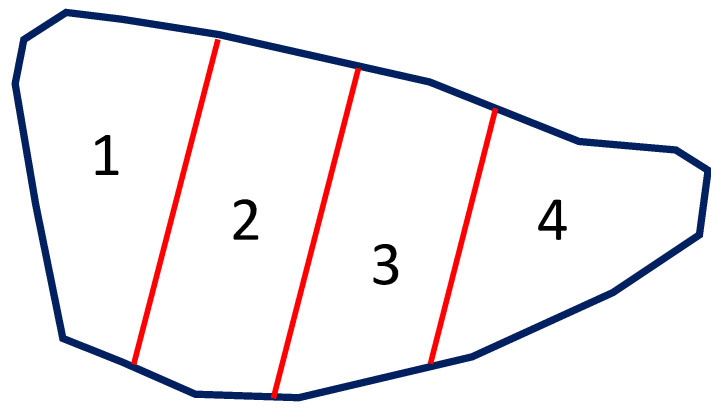
Schematic representation of the cardiac lobe of the porcine left lung. According to Madec’s grid, each pulmonary lobe is subdivided into 4 equal parts. The score is given through visual inspection and palpation, as follows: (0) no lesion; (1) EP-like lesion affecting <25% surface; (2) EP-like lesion affecting 25–49% of the surface; (3) EP-like lesion affecting 50–74% of the surface; (4) EP-like lesion affecting ≥75% of the surface.

Over time, several methods have been developed to score EP-like lesions [7], all sharing the same basic principle: the larger the EP-like lesions, the greater the economic loss. In Europe, “Madec’s grid” [8] is commonly used to score EP-like lesions in slaughtered pigs, as it is quite simple, fast, and compatible with the slaughter chain speed. Briefly, such a scoring method gives from 0 to 4 points for each lung lobe, regardless of the size of the same lobes (see Figure 1 for details).

Modified versions of Madec’s grid are often adopted, which consider the relative weight of each lobe [9] and/or rule out the accessory lobe from total scoring [10]. To date, the detection of EP-like lesions is based on visual inspection and palpation of the lungs. On the other hand, post mortem inspection has recently shifted towards visual-only examinations, aiming to reduce the risk of cross-contamination (EU Regulation 219/2014; EU Regulation 627/2019) [11].

Although useful, traditional lung lesion scoring methods (i.e., performed by skilled operators along the slaughter chain) are too expensive and time consuming to be consistently performed. Therefore, large amounts of data are lost and cannot be used to improve animal health and welfare. Artificial intelligence (AI)-based technologies, such as deep learning architectures (e.g., convolutional neural networks, CNNs), could properly fulfill such a task. During the last decade, ad hoc CNNs have been successfully trained to score pneumonia, pleurisy, and parasitic hepatitis (“white spots”) in slaughtered pigs [12,13,14]. One CNN has recently shown good performance when compared with the ability of veterinarians to detect and quantify EP-like lesions on digital pictures [14]. The present investigation aims to further evaluate this CNN by comparing its predictions with the absolute gold standard, that is, scores provided by a skilled operator who directly inspected and palpated the lungs along the slaughter chain.

## 2. Materials and Methods

### 2.1. Animals

A total of 441 lungs were investigated, taken from routinely slaughtered heavy pigs (approximate slaughter weight of 160 kg, average age of 9–10 months) and randomly distributed between right (n = 227) and left (n = 214).

As a routine, lungs were removed from the chest cavity and hung on a hook, along with other viscera (tongue, esophagus, heart, diaphragmatic muscle, liver, and kidneys). Pictures were taken along the slaughter chain by a veterinarian by means of a smartphone camera (Apple iPhone SE). More in detail, each lung was photographed from a distance of 0.5–1.0 m under field lighting conditions. The decision to take a picture of the right or left lung exclusively resulted from the accidental rotation of pluck. The lung was photographed in such a way that its external surface occupied most of the field of view. Consequently, the accessory lobe of the right lung was not visible. Lungs severely ripped because of chronic pleurisy were not included in this study.

Pictures were originally taken at 3072 × 2304 pixels of resolution and then re-sized at 400 × 300 pixels before being administered to the CNN, aiming to lighten its workload.

### 2.2. Scoring EP-like Lesions through Madec’s and Christensen’s Grids

Another skilled veterinarian examined the same lungs through careful visual inspection and palpation. A score was assigned to each lobe (including the accessory lobe) according to Madec’s grid. Moreover, such scores were multiplied by lobe-specific correction factors, according to Christensen et al. [9] (see Supplementary File S1 for details).

### 2.3. Scoring Predicted by a Previously Trained CNN

Details about the main features, training, and performances of the CNN have been previously published [14]. Briefly, it is a deep-learning-based model using a convolutional auto-encoder architecture based on U-Net. Such architecture has been extensively modified to accurately predict and segment the lung silhouette and EP-like lesions given an input image.

Collected images were provided to the CNN, which classified the input pictures as diseased or healthy based on the presence/absence of EP-like lesions. Whenever detected, the size of lesions was scored as a percentage of the entire lung surface.

### 2.4. Statistical Analysis

CNN performances were evaluated in terms of sensitivity, specificity, and correlation with the veterinarian’s scores, which represented the gold standard. Finally, the equivalence formula between the scores given by the operator and those provided by the CNN was computed.

## 3. Results

### 3.1. Scoring Provided by the Veterinarian through Madec’s and Christensen’s Grids

**Figure 2:**
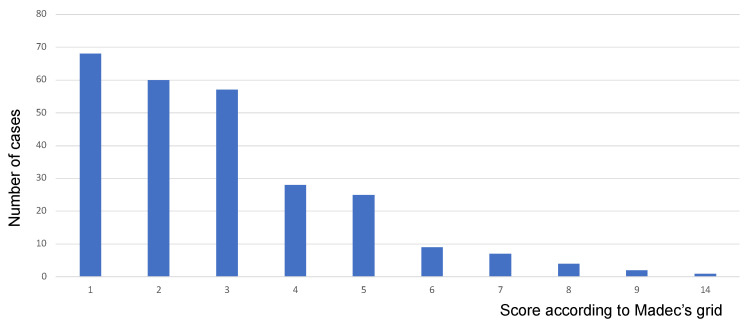
Most EP-like lesions were scored between 1 and 5. Only 7 lungs scored ≥ 8.

EP-like lesions were detected in 261 lungs (59.18%), while the remaining 180 lungs (40.81%) were considered healthy (i.e., score 0). In diseased lungs, scores ranged between 1 and 14, as reported in Figure 2. The accessory lobe was involved in 50 diseased right lungs, while in a single case, EP-like lesions exclusively affected the accessory lobe.

**Figure 3:**
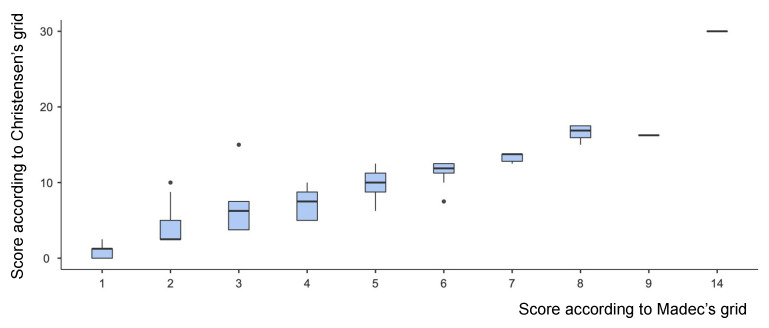
Madec’s vs. Christensen’s scores. Median values (horizontal thick lines), 25th to 75th percentile range (boxes), and extreme values (vertical thin lines) are reported. Dots indicate outliers (i.e., values falling outside the range). As clearly shown in this graph, a strong correlation exists between such scoring methods (Spearman’s coefficient = 0.962; *p* < 0.001).

Table 1 summarizes the veterinarian’s scores calculated according to Madec’s and Christensen’s grids, while Figure 3 graphically compares data obtained through such methods.

### 3.2. Scoring Predicted by the CNN

**Figure 4:**
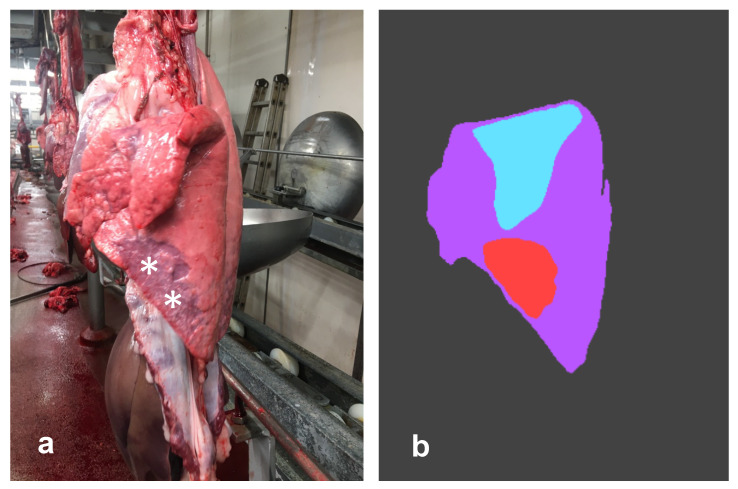
Left lung. The CNN erroneously detected a large lesion (**a**) along the ventral border of the diaphragmatic lobe (white asterisks). Although the exclusive involvement of that lobe is unusual, the morphological features of such an artifact might resemble EP. In such a case, palpation can be useful to confirm/rule out pneumonia. As shown in figure (**b**), the CNN correctly predicted the lung silhouette (purple) and the flipped lobe (light blue), while it detected a false EP-like lesion (red).

The CNN correctly predicted 172 out of 180 healthy lungs (specificity = 95.55%). Overall, the mean value given to healthy lungs was very close to 0 (0.06%). More in detail, the size of false EP-like lesions ranged from 1% to 9% of the entire pulmonary surface. In a single case, the error appeared quite serious, as the CNN mistakenly interpreted a large, partially collapsed area of the diaphragmatic lobe (see Figure 4).

**Figure 5:**
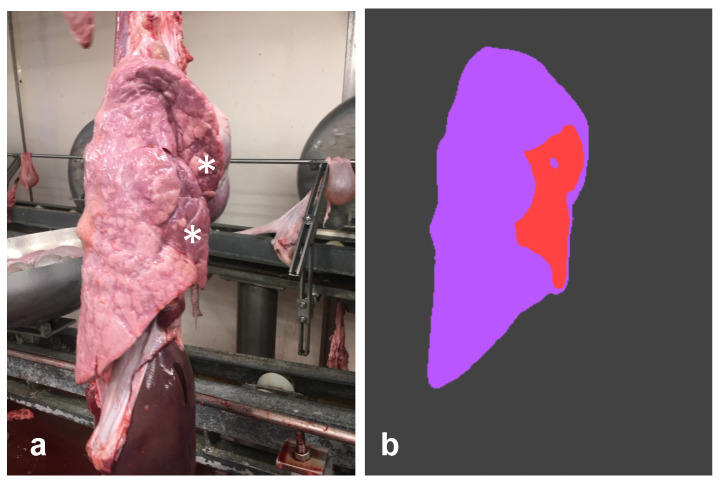
Right lung. The operator detected EP-like lesions (**a**) within the cranial and middle lobes (white asterisks, score 5 according to Madec’s grid). The CNN correctly predicted the lung silhouette (purple) and the EP-like lesion (red), scoring 15.82 (**b**).

Moreover, the CNN correctly recognized 222 out of 261 diseased lungs (sensitivity = 85.05%; see Figure 5 for details). Thus, EP-like lesions went unnoticed in 39 lungs.

**Figure 6:**
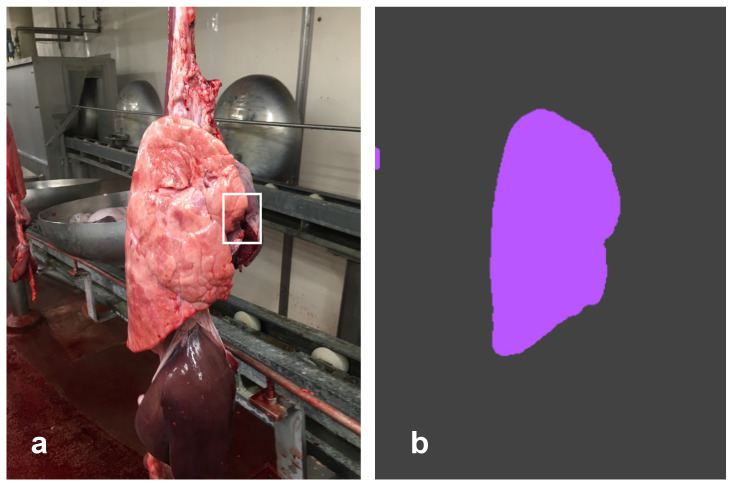
Right lung. The veterinarian detected a very small lesion (score 1 according to Madec’s grid) on the tip of the cranial lobe, partially overlapping with the cardiac muscle (**a**). The CNN correctly predicted the lung silhouette (purple), but it was unable to detect any lesion, no red spot being evident (**b**).

In 34 cases, the CNN error concerned small lesions, which had been scored 1 by the veterinarian (see Figure 6). In addition, the CNN mistakenly predicted five diseased lungs, which had been scored 2 (n = 2) and 3 (n = 3) according to Madec’s grid. In a single case, an EP-like lesion (score 2) affected only the accessory lobe. All lungs scoring ≥4 were correctly identified as affected by EP-like lesions.

Overall, the mean score predicted by the CNN was 4.5% (standard deviation = 0.08%; median = 0.5%). When computing only diseased lungs, the mean score was 9.6% (standard deviation = 0.094%; median = 7.4%).

### 3.3. Correlation among Scores Provided by the Veterinarian and Predicted by the CNN

**Figure 7:**
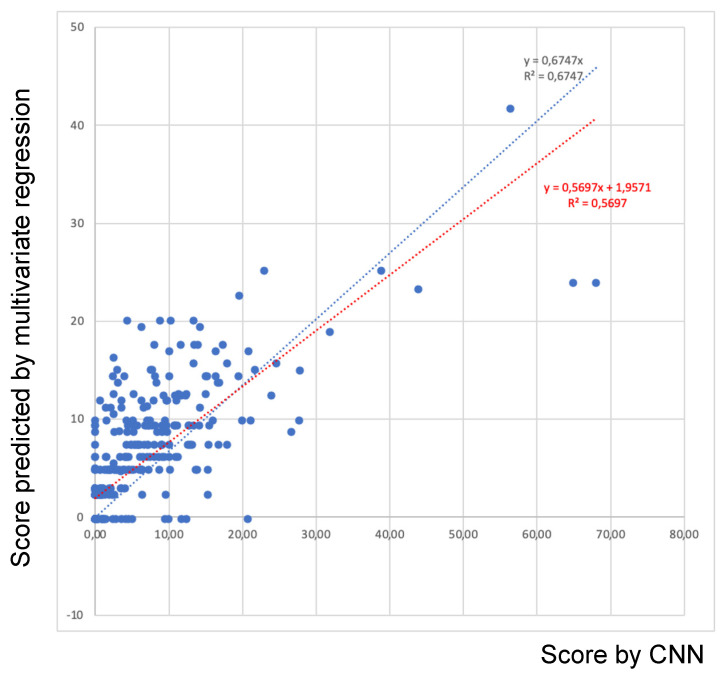
Plot of scores given by the CNN vs. scores provided by the statistical model (multivariate regression with Madec’s and Christensen’s values as independent variables). Red dotted line refers to linear model as calculated by the system, while blue dotted line was obtained posing the origin at the coordinates (0,0).

Madec’s scores for computing vs. ruling out the accessory lobe very strongly correlated (Spearman’s coefficient = 0.98, *p* < 0.001). Likewise, Christensen’s scores for computing vs. ruling out the accessory lobe very strongly correlated (Spearman’s coefficient = 0.99, *p* < 0.001). Scores assigned by the veterinarian according to Madec’s and Christensen’s grids (including the accessory lobe) and CNN predictions are graphically represented in Figure 7.

The CNN’s predictions strongly and significantly correlated with the veterinarian’s scores when computing both Madec’s and Christensen’s values (Spearman’s coefficient = 0.831, *p* < 0.01). The equivalence formula among different methods is reported below:

CNN’s prediction = 1.863 × (Madec’s score) + 0.528 × (Christensen’s score) − 0.208

The coefficient of determination (R^2^) was 0.6747, assessed by using a linear model and posing the origin at 0,0. Such a value is high and supports the reliability of the model. The confidence intervals (95%) for each parameter were determined as follows: 1.863 (1.001–2.270), 0.528 (−0.099–0.956), and 0.208 (−0.842–0.426).

## 4. Discussion

Virtual (e.g., computer vision system, CVS) and physical (e.g., robotics) branches of AI are deeply influencing most human activities, including biomedical sciences. A growing body of evidence indicates that AI-powered technologies can be successfully used to enhance disease diagnosis, management, and therapy in human medicine [15]. Likewise, although slower and less capital-intensive, AI-based technologies are also being employed in several fields of veterinary medicine, from livestock precision farming to advanced diagnostic imaging and from the epidemiology of livestock infections (e.g., vector-borne diseases) to animal welfare assessment [16].

Over the last few years, several research groups have attempted to develop CNNs to be used at slaughter. As reviewed by Sandberg et al. [17] and largely expected, the greatest efforts have been made in poultry and pig farming, which are the most intensive and technologically advanced ones. More in detail, 19 papers were about using a CVS to detect carcass surface contamination in chickens (n = 18) and bovines (n = 1), while 30 papers dealt with using a CVS to detect lesions during post mortem inspection in chickens (n = 26) and pigs (n = 4). Notably, current European legislation (EU Regulation 2017/625 and EC Regulation 2019/627) allows for the use of a CVS as a complementary tool in meat inspection, and such a technology (namely VetInspector) has already been approved in poultry meat inspection in Denmark [17].

Considering pigs, so far, the developed CNNs mainly focus on respiratory diseases (pneumonia and pleuritis), as they are the most relevant ones for farm profitability [12,13,14]. In this study, the CNN developed by Bonicelli et al. [14] was applied. This CNN demonstrated high accuracy rates, i.e., it was able to properly interpret digital images, almost like a skilled operator acting in front of a computer screen. Herein, we showed that the same CNN provides good results even when compared with the best scenario, i.e., scores given by a skilled operator after careful inspection and palpation.

Interestingly, Ghidini et al. [18] have shown that visual-only methods (namely Blaha’s scoring system) can be suitable, although they are less sensitive in scoring pneumonia in slaughtered pigs. Blaha’s and Madec’s methods demonstrated a strong and significant correlation (Spearman’s coefficient of 0.81, *p* < 0.001), their agreement being particularly good for medium-to-large-sized lesions [18]. Overall, such data match those reported in this study. As a matter of fact, the CNN did not detect several small foci of pneumonia, while it very seldom failed in cases of larger EP-like lesions. Worthy of note, the correlation between visual-only methods (i.e., the CNN’s predictions, Blaha’s method), on one side, and “traditional” methods (Madec’s and Christensen’s grids), on the other, was similar.

It is useful to retrospectively analyze the CNN’s mistakes, aiming to improve its performance. In the case of small lesions, errors usually resulted from the following factors: (1) size of pneumonic foci; (2) slight rotation of the affected lobe, which hid lesion; (3) the presence of small blood clots, which may adhere to the apex of the lobes (usually the middle lobes). Regarding larger lesions, the rare errors resulted from the partial overlapping of lobes and/or the presence of more pronounced artifacts. By definition, the CNN’s performance was lower when compared with the operator’s scoring, as the latter represents the benchmark. However, it is useful to point out that the CNN’s prediction is absolutely standardized and free from inter-operator variability, which is often difficult to estimate and manage.

Sibila et al. [19] developed a scoring system based on image analysis. Briefly, the dorsal side of both lungs was photographed, and lesions were delimited in the picture and quantified as a percentage of the entire lung surface. According to Garcia-Morante et al. [7], such method shows the “poorest” correlation (*r* = 0.725) with other scoring systems, maybe because it failed to count the accessory lobe. However, the relevance of the accessory lobe was not weighted in that paper [7]. In this study, the correlation between the CNN and traditional methods was not significantly influenced by the accessory lobe. Our findings indicate that the accessory lobe plays a marginal role and could be ignored, as some colleagues already do [10].

It should be noted that most scoring systems [8,9,20,21] are rather identical, representing minor “variations on a theme”, and thus it is not surprising to observe a very strong correlation among them. Image analysis (including AI-based methods) is conceptually different but not less suitable. Moreover, image analyses are more objective and repeatable in nature, while manual scoring is inherently dependent on the experience, attention, and sensitivity of the operator [7].

The development of CNNs is a pre-requisite to automatically score lesions, which would be economically viable in the medium-to-long term, thus making available the routine evaluation of all batches as well as the management of a huge amount of information. As a matter of fact, AI-based methods represent a unique tool to systematically analyze the health and welfare status of slaughtered animals, thus allowing robust and real-time feedback to stakeholders. Current technologies are already suitable to solve that task, as suggested by a preliminary trial that has been carried out to automatically score pleurisy under field conditions [22]. In our opinion, taking suitable photographs (i.e., of both lungs, each one in the center of the field of view and properly positioned) might be the toughest challenge to automatically score EP-like lesions, especially when operating in high-throughput abattoirs (slaughter chain speed > 6 pigs/min). In this respect, taking a single picture per pig could be a useful option, as suggested by some authors [7,23].

## 5. Conclusions

It is now widely accepted that AI will have a major impact on all human activities in the near future. Therefore, it appears of paramount relevance to understand AI strengths and weaknesses well. Overall, this study endorses the power of AI-based technologies in veterinary medicine to yield a substantial amount of data about animal health and welfare. Pig farming and high-throughput abattoirs could particularly benefit from automatized scoring systems, thus making AI-based technologies worthy of further attention and investment. We consider that the CNN tested herein already shows satisfactory performance, aiming to estimate the impact of EP on pig batches. Such performances are similar to visual-only methods and can improve over time through the continuing education of the CNN.

## Figures and Tables

**Table 1:** Summary of results obtained by applying traditional scoring methods. Data did show a non-Gaussian distribution in all considered scenarios (Shapiro–Wilk’s normality test; W value ranges between 0.773 and 0.865, *p* < 0.001).

	Madec’s Grid		Christensen’s Grid (%)	
	Considering All Lungs (n = 441)	Considering Only Diseased Lungs (n = 261)	Considering All Lungs (n = 441)	Considering Only Diseased Lungs (n = 261)
Median	1	3	0	2.625
Minimum	0	1	0	1.25
Maximum	14	14	13.75	13.75
25th percentile	0	2	0	2.5
75th percentile	3	4	1.25	3.75

## Data Availability

The datasets used and/or analyzed during the current study are available from the corresponding author upon reasonable request.

## Supplementary Materials

The following supporting information can be downloaded at: https://www.mdpi.com/article/10.3390/pathogens12121460/s1, Table S1: Lobe-specific capacity according to Christensen et al. (1999).
