# Supplementary material for: Scoring Enzootic Pneumonia-like Lesions in Slaughtered Pigs: Traditional vs. Artificial-Intelligence-Based Methods
Source: Pathogens. 2023 Dec 17;12(12):1460. doi: 10.3390/pathogens12121460 (PMC10747234; doi:10.3390/pathogens12121460)
Supplement: Supplementary file 1 [file pathogens-12-01460-s001.zip › pathogens-2730855-supplementary.pdf]

Table S1: Lobe-specific capacity. Considering that lung lobes are not all the same size, Christensen *et al*. (1999) assigned a specific weight to each lobe (expressed as percentage of the entire pulmonary capacity), as reported in the following Table.

**Lung lobe**	**Percentage of the entire pulmonary capacity, as assigned by Christensen *et al.* (1999)**
Cranial lobe of the left lung	5
Middle (cardiac) lobe of the left lung	6
Diaphragmatic (caudal) lobe of the left lung	29
Cranial lobe of the right lung	11
Middle lobe of the right lung	10
Diaphragmatic (caudal) lobe of the right lung	34
Accessory lobe	5
